# Evaluation of a Filtering Facepiece Respirator and a Pleated Particulate Respirator in Filtering Ultrafine Particles and Submicron Particles in Welding and Asphalt Plant Work Environments

**DOI:** 10.3390/ijerph18126437

**Published:** 2021-06-14

**Authors:** Aniruddha Mitra, Atin Adhikari, Clinton Martin, Gracia Dardano, Pascal Wagemaker, Caleb Adeoye

**Affiliations:** 1 Department of Mechanical Engineering, Georgia Southern University, P.O. Box 8046, Statesboro, GA 30460, USA; gd01213@georgiasouthern.edu (G.D.); pw01801@georgiasouthern.edu (P.W.); 2 Jiann-Ping Hsu College of Public Health, Georgia Southern University, P.O. Box 8015, Statesboro, GA 30460, USA; aadhikari@georgiasouthern.edu (A.A.); ca13007@georgiasouthern.edu (C.A.); 3 Department of Civil Engineering & Construction Management, Georgia Southern University, P.O. Box 8077, Statesboro, GA 30460, USA; cdmartin@georgiasouthern.edu

**Keywords:** ultrafine particle, welding, asphalt plant, filtration, respirator, occupational safety, personal protective equipment

## Abstract

Manufacturing sites, such as welding, casting, and asphalt production (fumes), generate vast numbers of ultrafine particles of <0.1 µm in size and submicron particles close to the ultrafine range (0.1–0.5 µm). Although cumulative masses of these particles are negligible in comparison to the larger particles, the health effects are more severe due to the higher penetration in the human lower respiratory tract, other body parts crossing the respiratory epithelial layers, and the larger surface area. This research investigates the effectiveness of two common commercially available N95 filtering facepieces and N95 pleated particulate respirator models against ultrafine and submicron particles. Two specific types of respirators, the N95 filtering facepiece and the N95 pleated particulate models, in both sealed and unsealed conditions to the manikin face, were tested at various commercial and academic manufacturing sites, a welding and foundry site, and an asphalt production plant. Two TSI Nanoscan SMPS nanoparticle counters were used simultaneously to collect data for particles of 10–420 nm in size from inside and outside of the respirators. While one of them represented the workplace exposure levels, the other one accounted for the exposure upon filtration through the respiratory surfaces. The results showed the particles generated by these manufacturing operations were mostly within the range of from 40 to 200 nm. Results also indicated that while the percentage of filtration levels varied based on the particle size, it remained mostly within the desired protection level of 95% for both of the N95 respirator models in sealed conditions and even for the N95 pleated particulate model in the unsealed condition. However, in the case of the N95 filtering facepiece model, unsealed respirators showed that the percentage of penetration was very high, decreasing the protection levels to 60% in some cases. Although the number of workplace airborne particle levels varied considerably, the filtration percentages were relatively consistent.

## 1. Introduction

Ultrafine particles are a particulate of matter lying within 1 and 100 nanometers in size and are usually generated either naturally or created with engineering methods and procedures. Ultrafine particles are released naturally during events such as forest fires or by the industry during combustion processes. Natural ultrafine particles include ashes, viruses, and smoke; engineered ultrafine particles are produced in processes such as asphalt and concrete mixing, welding, cosmetic manufacturing, as well as others [1]. Given their size, ultrafine particles are difficult to measure in work environments, and their harmful effects can be overlooked.

The theory of Brownian Motion describes the motion of ultrafine particles as random and with constant collisions that lead to high values of momentum [2]. Given their random nature, it has been increasingly hard to analyze the behavior and regulate their effects on human health [3]. Multiple studies have been performed to evaluate and understand the nature and behavior of ultrafine particles. It has been concluded that there is a strong presence of ultrafine particles in environments that interact with welding fumes [4,5]. The conclusion is very relevant because the composition of fumes involves particles rich in metals that are dangerous to humans [4,5]. The health effects of ultrafine particles have been reviewed in detail by Schraufnagel [6]. This review suggested that characteristics such as particle material, mass, size, and surface are critical to understanding the health effects of ultrafine particles [6]. Strong correlations between the particle size, surface, and respiratory and cardiovascular complications from inhaling particles, were reported [7,8,9]. Therefore, ultrafine particles are considered more toxic than fine or coarse particles [10,11] because of their cumulative large particle surface area carrying large amounts of absorbed pollutants [6] including the reactive substances like metal and concrete mixes, which may increase the mortality resulting from the interaction with the particles [5,12].

Few research studies have been performed on the effectiveness of face masks and air filters on filtering out ultrafine particles. While facepiece respirators and filters have strict standards for micro and other, larger-sized particles, the protection and filtration devices used currently do not have standards for preventing ultrafine particles from entering the human body. The highest concentrations of ultrafine particles are typically found in welding shops, machine shops, and the metal industries [6]. Studies have shown that inhaling these ultrafine particles can result in health risks, such as inflammation and toxicity, and that these risks are more dependent on the specific surface area than the total mass [13]. Since ultrafine particles have a larger relative surface area than larger-sized particles, they pose a greater risk to human health. Their smaller sizes also mean that they are more difficult to filter out and can penetrate further into the human lower respiratory tract. It is critical to understand the behavior of these particles. Ultrafine particles have been shown to pose an even greater inflammatory response potential than more common fine particles [14]. Therefore, it is essential to understand the process of filtering out ultrafine particles during processes such as welding.

The ready-mix concrete (RMC) industry, which is one of the most essential sub-sectors in modern construction, is responsible for producing construction materials which are crucial for building large engineering structures including roads, bridges, homes, and high-rises. Combining fine and coarse aggregates, cement and water, creates RMC, there are many advantages of using RMC such as its speedy construction through computer programmed delivery at site, consistency of quality through accurate computerized control of sand aggregates and water as per mix designs, and ability to minimize cement wastage due to bulk handling, reducing the labor cost, and economy regarding the use of raw materials [15]. Besides all these advantages, the RMC industry in the US, however, still suffers from safety problems, regardless of its leading the world in RMC production, with over 5000 plants and 68,500 trucks [16]. The occupational safety problems are related to many tasks in an RMC plant involving the operation of machines and the assembly of tools, such as mixers, cement batchers, aggregate batchers, conveyors, radial stackers, aggregate bins, cement bins, heaters, chillers, and cement silos. These operations partially take place in the production area, where RMC is produced and loaded into mixer trucks, and exposes RMC workers to various safety hazards. Potential hazards for workers in these work settings include: eye, skin, and respiratory tract irritation from exposure to cement dust, overexertion and awkward postures (ergonomics), slips, trips, falls, chemical burns from wet concrete, loss of stability, cutting, severing, and hazards generated by vibration and radiation. The sources of these hazards are usually derived from system failure, inadequate safety guards on equipment, inadequate lockout/tag-out systems on machinery (mechanical and electrical hazards), ejection of parts or material, shearing hazards generated by noise, stabbing, puncture, friction, abrasion, high-pressure fluid injection, or combined hazards [17,18]. Some of the RMC works potentially involve silica exposure due to the silica-containing concrete composition of RMC in the range of 10–20%. Silica is an ingredient of RMC that becomes airborne during jack-hammering operations. Exposure to silica causes silicosis, a disabling, nonreversible, and sometimes fatal lung disease caused by overexposure to respirable crystalline silica. More than one million U.S. workers are exposed to crystalline silica, and each year more than 250 die from silicosis [19]. There is no cure for the disease, but it is 100 percent preventable if employers, workers, and health professionals work together to reduce exposures. Therefore, ventilation of the working areas, adequate cleaning of the dust, and provision of safe working conditions are recommended for reducing the risk of silica exposure. RMC plant operators are also exposed to dust containing cement during the loading process at the power plant. Operators may be exposed to skin contact with concrete mixtures and additives containing irritants. Cement products are inherently high-risk products. It is known that wet concrete reacts with skin, natural moisture, and mucus layers of the eye. In addition, concrete contains a chrome component which is a strong irritant. These materials can cause skin irritation and allergic reactions.

According to OSHA, more than 250,000 people work in concrete manufacturing in the US [20]. Unfortunately, of the hundreds of thousands who work in concrete product manufacturing, tens of thousands have experienced a job-related injury, illness, or death. Over 10 percent of those workers—approximately 28,000—experienced a job-related injury or illness and 42 died in just one year [17]. The 2013 Bureau of Labor Statistics data reported injuries in the RMC industry with an incidence rate of 4.8 [21]. It was observed that there is a lack of a holistic assessment of hazards and risks that might occur during the manufacturing and handling of RMC. Only guidelines and manuals printed by associations such as OSHA, ACPA, and NSCSA are available to build safety awareness for RMC producers [17,22,23]. Actual field research on RMC occupational health and safety-related issues is still inadequate.

There have been several studies on the effects of welding on the environment. Cho et al. [24,25] use aerosol to evaluate the efficiencies of various particulate respirators. There has even been work on the possible leakage in the respirator which shows that the effectiveness of the filter is a function of the particle size [26]. Extensive review studies have been carried out to evaluate the effectiveness of the commonly used N95 respirators in the recent past [27]. Additionally, a hygiene database of exposure has been created specifically for asphalt industry workers to track their overall cumulative health effects [28].

In this study, the exposure to ultrafine particles that occurs during welding activities and regular activities of asphalt plants was measured and compared to exposure levels after filtration through a facepiece filter. Commercially available N95 filtering facepiece and N95 pleated particulate respirator model were used as filters, in both sealed and unsealed conditions. Die casting, MIG welding, and stick welding sites were analyzed. Measurements were taken from approximately 1 m away from the welding spot to simulate the exposure of a welder, and from approximately 2–3 m away to simulate the exposure levels of an observer. Two 3910 NanoScan SMPS Nanoparticle Sizer devices were used simultaneously. One NanoScan measured the unfiltered nanoparticle exposure levels, while the other NanoScan measured the exposure levels after filtration. This research aims to compare the effectiveness of two commercially available facepieces against ultrafine and submicron particles during different manufacturing processes.

## 2. Materials and Methods

To measure the effectiveness in filtration of the respirators, a field-compatible testing setup was developed that allowed for running the air sample collection and parallel evaluation of respirators at numerous testing locations simultaneously. A similar setup was used by two of the current authors in evaluating the N95 facepiece at construction sites [29]. The setup included two NanoScan SMPS ultrafine particle monitors (model: 3910, manufacturer: TSI). These nanoparticle scanning devices have a 60-s scan time, inhaling and analyzing 1 cubic centimeter of air each time. The scanner detects particle sizes ranging from 10 nm to 420 nm. It then distributes the particles in 13 different bin sizes that are logarithmically scaled. The medians of these 13 bin sizes in nm are 11.5, 15.4, 20.5, 27.4, 36.5, 48.7, 64.9, 86.6, 115.5, 154, 205.4, 273.8, and 365.2. It repeats itself every minute. Hence, a ten-minute-long test consists of ten cycles with ten datapoints for each bin. At each location, for every combination of parameters, at least ten such datapoints are collected.

Two work environments with possible high concentrations of ultrafine particles, such as welding work sites and workshops, and asphalt plants, were targeted to determine the effectiveness in ultrafine particle and submicron particle filtration by the respirators commonly used by workers. The setup included:

- Two NanoScan SMPS 3910 machines, used to measure ultrafine particle concentration;
- Three sampling probes, used to maintain airflows;
- N95 respirators, used to determine their ultrafine particle filtration effectiveness;
- A foam manikin head to simulate human interaction with the environment;
- 85 L per minute air pump used to simulate human breathing effects;
- A portable stand and cart used to transport testing configuration to different locations.

**Figure 1:**
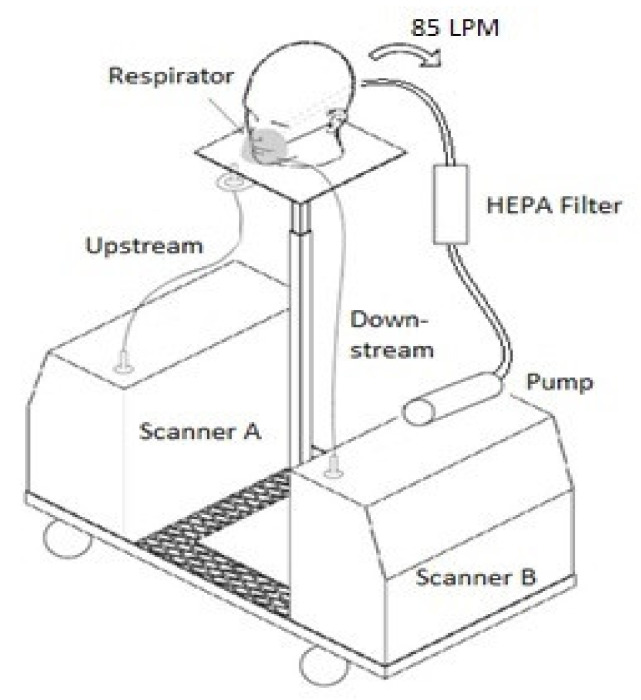
Schematic diagram of a respirator testing setup.

The setup was used in construction sites to simulate a construction worker and their exposure to ultrafine particles. A schematic diagram for the respirator testing setup is displayed in Figure 1. It is important to note that two NanoScan SMPS 3910s were used simultaneously in the data collection process. One machine measured the unfiltered, upstream airflow outside of the respirator. The other machine measured the filtered, downstream airflow inside of the respirator simultaneously. Airflow was created by an 85 L/min air pump, simulating the airflow pulling a similar amount of air into the respirator to that drawn by a human while breathing normally. According to the National Institute for Occupational Safety and Health (US NIOSH) N95 Filtering Facepiece Respirator (FFR) certification method, an N95 FFR sealed onto a plate should be tested against the airflow of 85 L/min [30]. Therefore, we have considered this airflow in our experimental setup. The setup was designed to be portable so that it could be transported it to different spots within a location and moved from one location to another. Therefore, a utility cart was developed with a built-in stand that held the two NanoScan machines and the manikin heads with adjustable heights.

**Figure 2:**
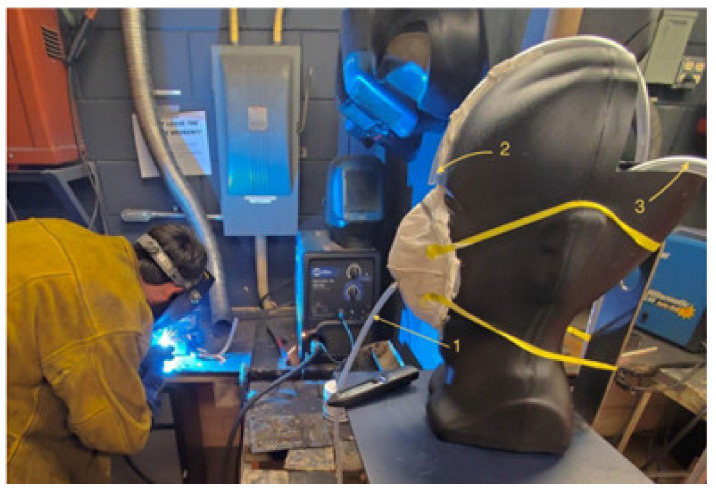
Respirator testing setup with numbered probes.

In Figure 2, an image of the actual setup in a welding location is displayed. The three sampling probes have been numbered to explain their functions. Probe 1 directed filtered airflow from behind the respirator into NanoScan Scanner B. Probe 2 directed unfiltered airflow from directly in front of the respirator into NanoScan Scanner A. Finally, Hose 3 traveled through the nose and from the back of the head and connected to a vacuum pump, simulating the workings of the lungs pulling in air from inside the respirator. The pump drew air at a consistent rate of 85 L/min, which simulates normal human breathing conditions in work environments.

During the experiment, sampling probes acquired data indicating the level of nanoparticle concentration on both sides of the respirator simultaneously. Data were generated from measured nanoparticle concentrations at welding shops at Georgia Southern University as well as local industrial sites. More specifically, the sites analyzed in this project were:

- Industrial Manufacturing Plant located in South Georgia;
- Georgia Southern University welding shop;
- Asphalt Production Plant located in South Georgia.

The testing setup at the sites mentioned above is displayed in the Supplementary Materials in Figures S1–S3..

**Figure 3:**
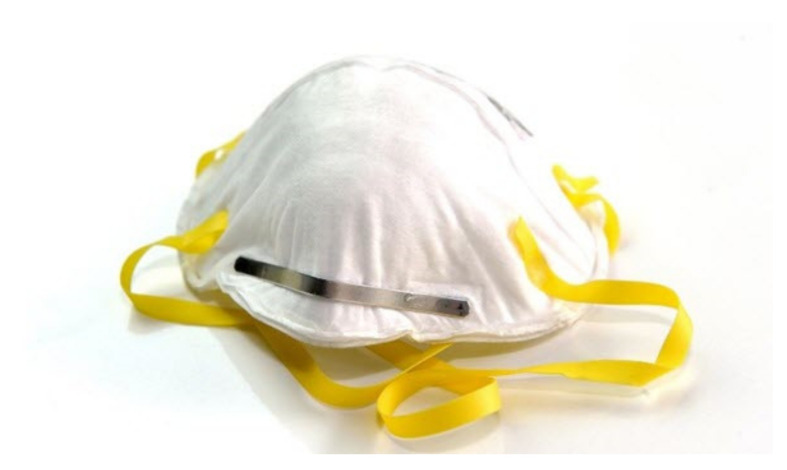
N95 non-pleated respirator.

**Figure 4:**
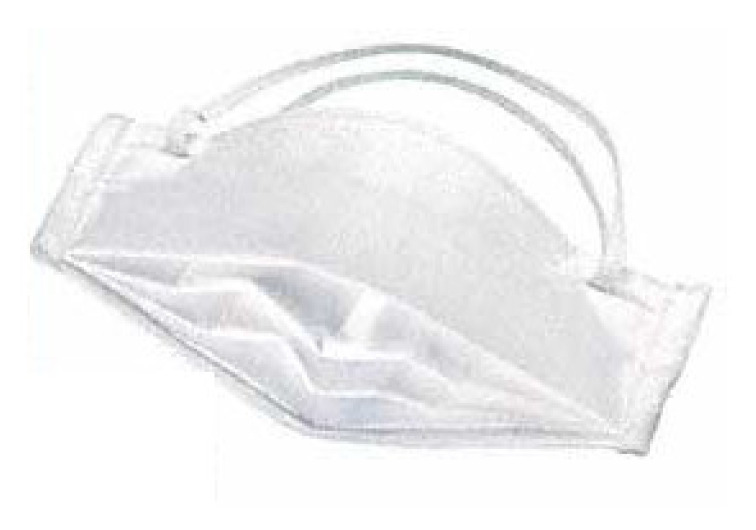
N95 pleated respirator.

The respirators being analyzed in this process are two N95 respirators, one pleated and one non-pleated. Respirators were tested both unsealed and sealed to the manikin’s face. The N95 pleated and non-pleated respirators are displayed in Figure 3 and Figure 4 below, respectively.

## 3. Results

Vast quantities of data, consisting of 90 samples of data for 13 bin sizes, were obtained from the two NanoScan machines in which there were roughly 600 datapoints for each of the machine’s trials. The preliminary data were analyzed and mean $\bar{x}$, and standard deviations σ, were calculated for each setup. The data that fell outside of the plus minus three sigma range were discarded and the new mean and standard deviations were calculated. This cyclic process was terminated when all the data fell within the six sigma range.

This refined data are presented based on the industrial sites where the experiments were conducted. Three different industrial sites in the southern region of Georgia (USA) were used: an engineering production company, Georgia Southern University’s Welding Shop, and an Asphalt Production Plant. Moreover, this can be further divided based upon the areas within the industrial sites themselves. For instance, three different areas were analyzed for the engineering manufacturing site: Die Casting Facility, Direct Airflow from MIG Welding and Observer Position Stick Welding. In a similar fashion, the tests were run in two locations for the Asphalt Manufacturing Plant.

The data were further processed, and filtration ratios were calculated for each particle size level. Average filtration ratios were also calculated for each respirator at its different settings. The results were tabulated and graphed for different locations at the industrial site, Die Casting Facility, Direct Airflow from MIG Welding, and Observer Position Stick Welding. For the Die Casting Facility and Direct Airflow from MIG welding, no data were removed in the six sigma data elimination process. For the Observer Position Stick Welding, only 1% of the data were removed. For the Carruth Welding Shop, 5.8% of data were removed. The Asphalt Plant had 25.5% of data removed during the cyclic process.

Except for Georgia Southern University’s Welding Shop, there was no significant detection of particles beyond the size range of 150 nm.

### 3.1. Engineering Manufacturing Site

#### 3.1.1. Die Casting Facility

**Figure 5:**
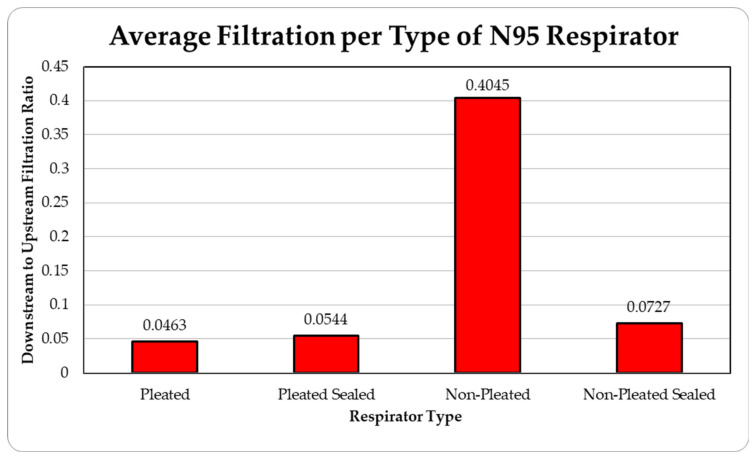
Average filtration ratios of total particles per type and mass at the die casting facility.

**Figure 6:**
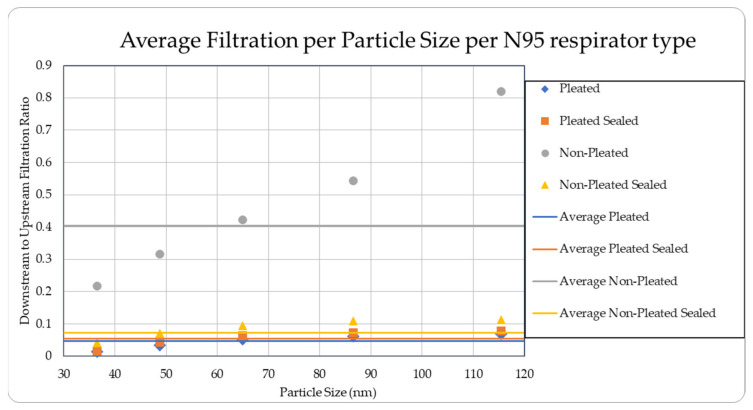
Average filtration ratios per type and per particle size at the Die Casting Facility.

The data were tabulated as shown below (Table 1, Figure 5 and Figure 6).

#### 3.1.2. Direct Airflow from MIG Welding

**Figure 7:**
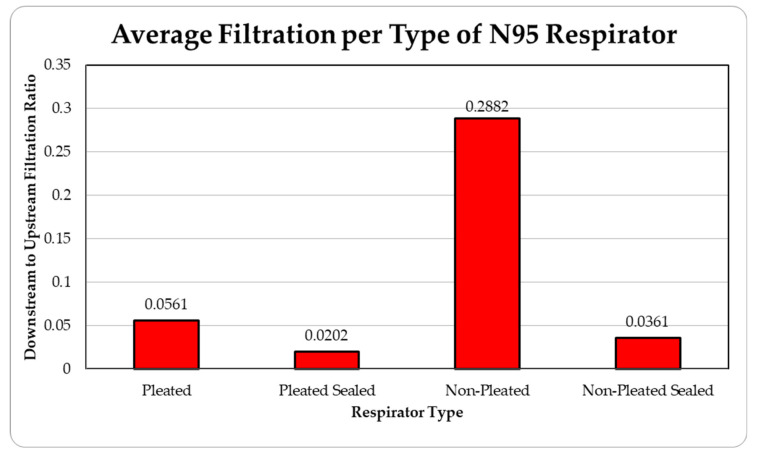
Average filtration ratios of total particles per type of respirator at the direct airflow from MIG Welding.

**Figure 8:**
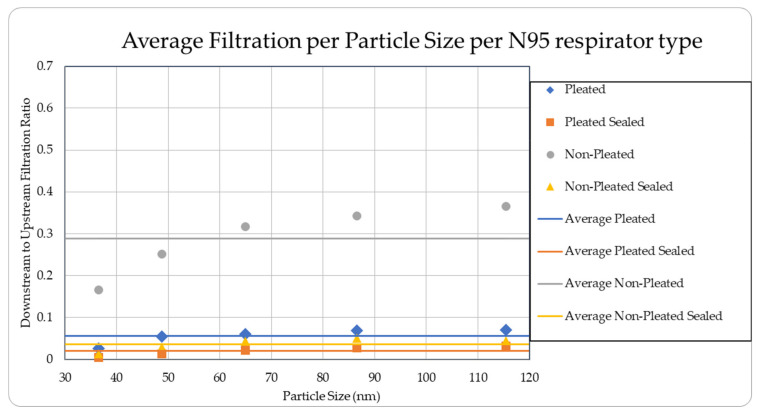
Average filtration ratios per type and per particle size from MIG Welding.

The data were tabulated as shown below (Table 2, Figure 7 and Figure 8).

#### 3.1.3. Observer Position Stick Welding

**Figure 9:**
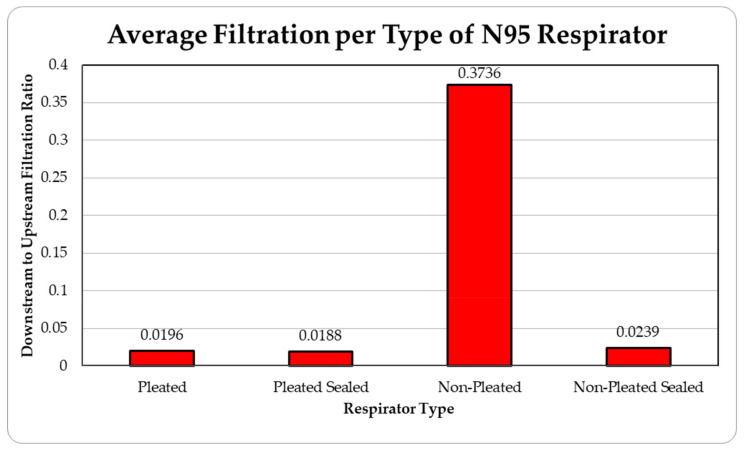
Average filtration ratios of total particles per type of respirator at the Observer Position Stick Welding.

**Figure 10:**
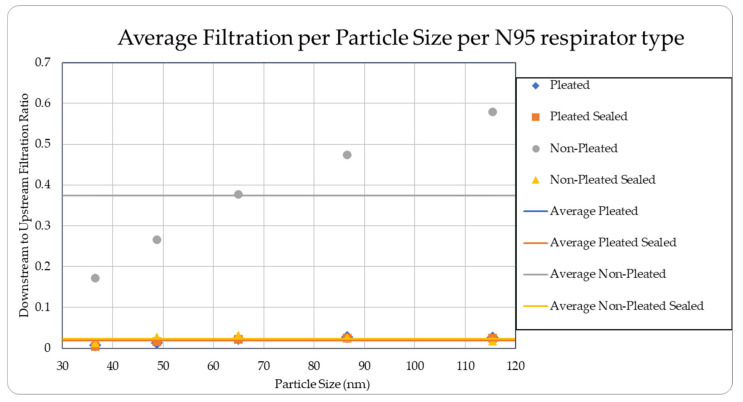
Average filtration ratios per particle size at the Observer Position Stick Welding.

The data were tabulated as shown below (Table 3, Figure 9 and Figure 10).

### 3.2. Georgia Southern University’s Welding Shop

**Figure 11:**
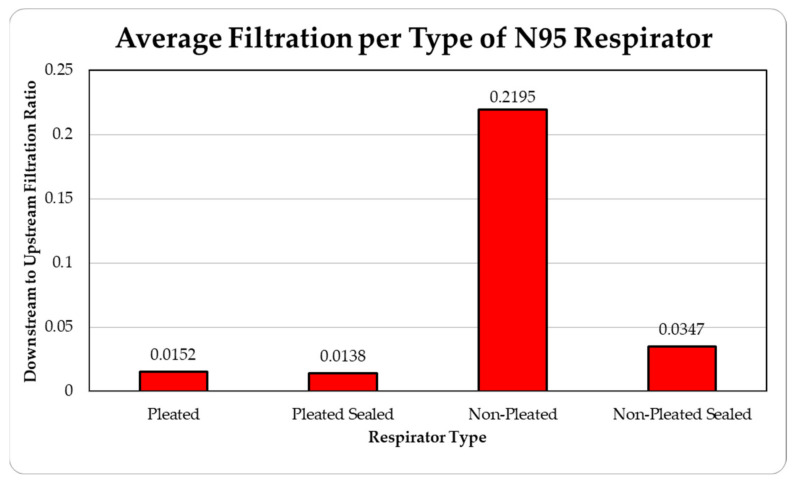
Average filtration ratios of total particles per type of respirator at the Carruth Welding Shop.

**Figure 12:**
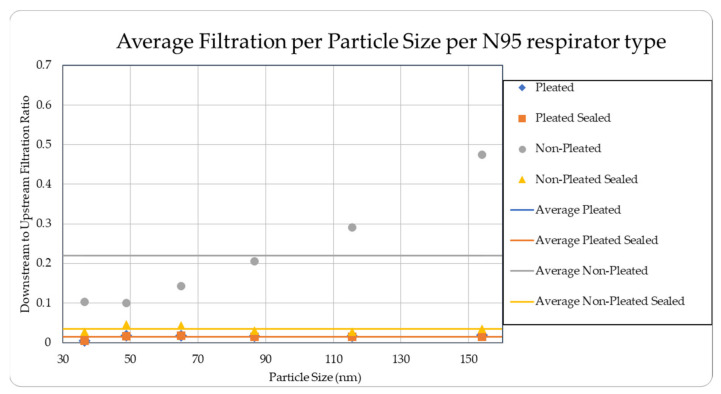
Average filtration ratios per particle size at the Carruth Welding Shop.

The data were tabulated as shown below (Table 4, Figure 11 and Figure 12).

### 3.3. Asphalt Plant

The data were collected at two different locations and were subsequently combined.

The N95 respirators analyzed were:

- Pleated sealed;
- Non-pleated;
- Non-pleated sealed.

**Figure 13:**
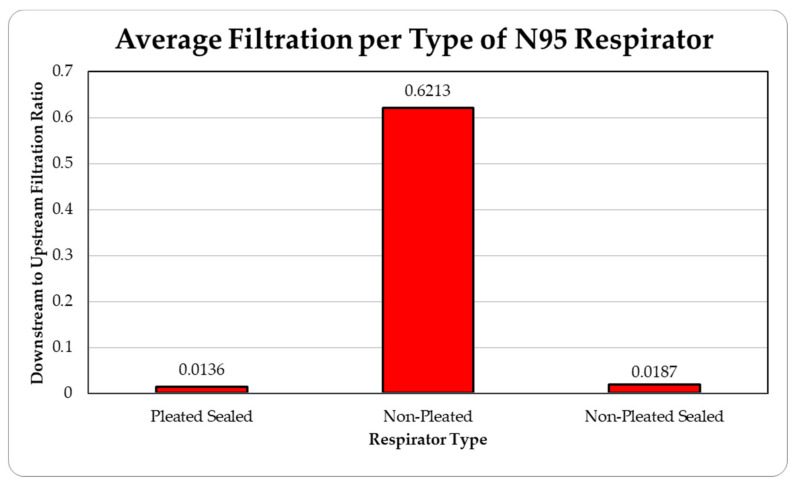
Average filtration ratios of total particles per type of respirator at the Asphalt Plant.

**Figure 14:**
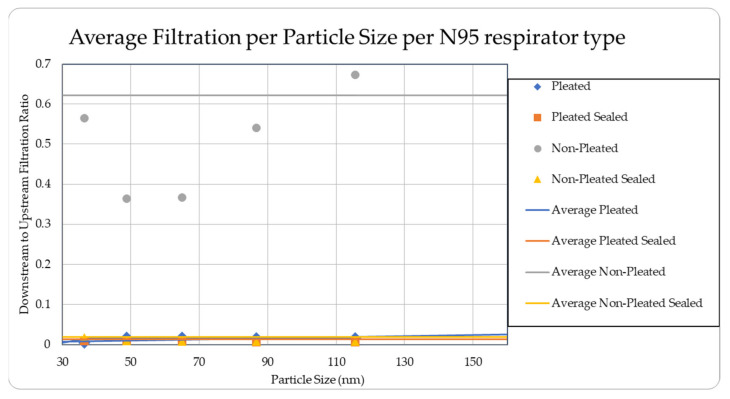
Average filtration ratios per particle size and type at the Asphalt Plant.

The data were tabulated as shown below (Table 5, Figure 13 and Figure 14).

## 4. Discussion

The level of concentration of particles over the size of 150 nm was low due to many zero values which prevented drawing any statistically significant inference, and hence were ignored in the result section. These observations were made for all three site locations. Since the ambient upstream nanoparticle concentration is constantly changing due to the randomness of nanoparticle movements and external influences, no trial experienced identical ambient nanoparticle concentrations. Due to this, trials were performed with two nanoparticle scanning devices, measuring ambient and filtered airflows simultaneously. This enables the evaluation of appropriate filtration rates of the facemasks without the influence from fluctuating ambient conditions. To provide a sense of the nanoparticle exposure in different working environments, average concentrations are provided in Table 1, Table 2, Table 3, Table 4 and Table 5. The results are also graphically presented in Figure 5, Figure 6, Figure 7, Figure 8, Figure 9, Figure 10, Figure 11, Figure 12, Figure 13 and Figure 14. Figure 5, Figure 7, Figure 9, Figure 11, and Figure 13 show the average filtration rates at different particle generation sources and locations for various filtering facepieces (pleated or non-pleated) and conditions (sealed or unsealed). Figure 6, Figure 8, Figure 10, Figure 12, and Figure 14 show corresponding details concerning the demographic of the particle sizes.

For a pleated facepiece, whether it is sealed or unsealed, the level of filtration is quite effective. It has always been within 5% for the entire spectrum of the particle size range. The variations are quite random and not statistically significant. So, for a pleated facepiece, whether it is sealed or not, obeys anticipated filtration by an N95 respirator (95% protection for the most penetrating 0.3 µm particles) based on our collected data. For the unpleated sealed facepiece, the effect is approximately the same. It is consistently less than 5% for the entire spectrum of particle sizes for all sites. However, it has been observed that, for an unsealed unpleated facepiece, the filtration level is poor, probably due to the loose fit on the manikin face surfaces. In most cases, the protection level was below the limit of 95%. It starts just above 10% for the smaller particle size and increases with the particle size and reaches to as high as 60% in some cases. This trend was noticed for all the sites except for the asphalt plant, where there is no clear trend. For the particle size of 27.4 nm, the percentages of filtration levels were too high and are not reported.

There are quite a few parameters that can affect the effectiveness of filtration by the facepiece, such as size of the particle, electrostatic attractions, properties of the fibers used in the respirator and particles, airflow patterns through the facepiece, thermal rebound, relative humidity, loading time, and filter chemical composition [31,32]. The current research only investigates two of these parameters, particle sources and particle size. However, considering other uncertainties, the results are quite consistent and exhibit a clear trend. The current results seem to be in general agreement with the recent results published in the literature, where percentage penetrations were found to be within 6% in all the cases under study covering the time span from 2000 to 2016 [27]. Future research will involve the effects due to the change in some of the parameters, such as different brands of commercially available pleated and unpleated facepieces.

## 5. Conclusions

An experimental study was undertaken to measure the effectiveness of commercially available filtering facepieces against ultrafine particles and submicron particles of up to 150 nm in size. The experimental sites were chosen where significantly high levels of ultrafine particles were generated due to manufacturing activities, such as welding, asphalt manufacturing, and casting. The results indicate that, for particle sizes between 10 nm to 150 nm, a pleated facepiece respirator is quite effective at maintaining the required 95% filtration standard for N95 respirators. However, for the unpleated facepiece, the results show that the filtration level is poor and certainly does not maintain the standard. Results also indicate that this type of facepiece is less effective in resisting larger particle sizes. Further investigation of different commercial brands of facepieces is recommended.

## Figures and Tables

**Table 1:** Results of average downstream to upstream filtration ratios per type, particle size, and per respirator type at the die casting facility.

Respirator Type	Particle Median Size (nm)					Overall Total Particles	Standard Deviation
	36.5	48.7	64.9	86.6	115.5		
Pleated	0.01383	0.03488	0.05182	0.06178	0.06942	0.04634	0.01282
Pleated Sealed	0.01639	0.04259	0.06242	0.07268	0.07796	0.05441	0.00686
Non-Pleated	0.21791	0.31613	0.42270	0.54403	0.81995	0.40452	0.09403
Non-Pleated Sealed	0.04107	0.07030	0.09478	0.10821	0.11280	0.07272	0.00930
Ambient Condition (#count/cc)	51,415	57,761	52,272	38,490	19,834	219,774	16,202

**Table 2:** Results of average downstream to upstream filtration ratios per type, particle size, and per respirator type at the Direct Airflow from MIG Welding.

Respirator Type	Particle Median Size (nm)					Overall Total Particles	Standard Deviation
	36.5	48.7	64.9	86.6	115.5		
Pleated	0.02603	0.05422	0.06104	0.06931	0.07005	0.05613	0.01825
Pleated Sealed	0.00457	0.01375	0.02266	0.02823	0.03166	0.02017	0.00831
Non-Pleated	0.16569	0.25111	0.31648	0.34219	0.36562	0.28822	0.07799
Non-Pleated Sealed	0.01337	0.02863	0.04320	0.05019	0.04486	0.03605	0.00589
Ambient Condition (#count/cc)	18,879	19,097	17,021	13,631	8680	77,309	4906

**Table 3:** Results of average downstream to upstream filtration ratios per type, particle size, and per respirator type at the Observer Position Stick Welding.

Respirator Type	Particle Median Size (nm)					Overall Total Particles	Standard Deviation
	36.5	48.7	64.9	86.6	115.5		
Pleated	0.00759	0.01392	0.02319	0.02730	0.02595	0.01959	0.00862
Pleated Sealed	0.00530	0.01577	0.02264	0.02264	0.02517	0.01881	0.00534
Non-Pleated	0.17206	0.26575	0.37700	0.47382	0.57932	0.37359	0.05245
Non-Pleated Sealed	0.01347	0.02819	0.03241	0.02701	0.01823	0.02386	0.00251
Ambient Condition (#count/cc)	16,068	18,441	20,243	20,949	17,860	93,564	8399

**Table 4:** Results of average downstream to upstream filtration ratios per type, particle size, and per respirator type at the Carruth Welding Shop.

Respirator Type	Particle Median Size (nm)						Overall Total Particles	Standard Deviation
	36.5	48.7	64.9	86.6	115.5	154		
Pleated	0.00515	0.01777	0.01734	0.01627	0.01612	0.01862	0.02079	0.00697
Pleated Sealed	0.00484	0.01640	0.01757	0.01521	0.01426	0.01463	0.01521	0.00473
Non-Pleated	0.10275	0.10021	0.14261	0.20520	0.29152	0.47497	0.21954	0.06592
Non-Pleated Sealed	0.02801	0.04601	0.04261	0.03042	0.02752	0.03388	0.01382	0.01147
Ambient Condition (#count/cc)	5280	9811	17,985	25,988	25,861	16,080	101,007	15,259

**Table 5:** Results of average downstream to upstream filtration ratios per type, particle size, and per respirator type at the Asphalt Plant.

Respirator Type	Particle Median Size (nm)							Overall Total Particles	Standard Deviation
	20.5	27.4	36.5	48.7	64.9	86.6	115.5		
Pleated Sealed	0.01628	0.03852	0.00926	0.00915	0.00865	0.00681	0.00675	0.01363	0.01340
Non-Pleated	0.58545	*	0.56447	0.36353	0.36664	0.54000	0.67301	0.53063	0.42606
Non-Pleated Sealed	0.01546	0.06560	0.01822	0.00863	0.00730	0.00762	0.00787	0.01867	0.01563
Ambient Condition (#count/cc)	436	484	328	385	759	1225	1390	5010	1043

* filtration ratio greater than 1 is omitted from the table.

## Data Availability

All collected data are presented in the article.

## Supplementary Materials

The following are available online at https://www.mdpi.com/article/10.3390/ijerph18126437/s1, Figure S1: Industrial manufacturing plant with testing setup, Figure S2: Georgia Southern University Welding Shop with testing setup, Figure S3: Asphalt Production Plant with testing setup.
