# Supplementary material for: Evaluation of a Filtering Facepiece Respirator and a Pleated Particulate Respirator in Filtering Ultrafine Particles and Submicron Particles in Welding and Asphalt Plant Work Environments
Source: Int J Environ Res Public Health. 2021 Jun 14;18(12):6437. doi: 10.3390/ijerph18126437 (PMC8296285; doi:10.3390/ijerph18126437)
Supplement: Supplementary file 1 [file ijerph-18-06437-s001.zip › ijerph-1199641-supplementary.pdf]

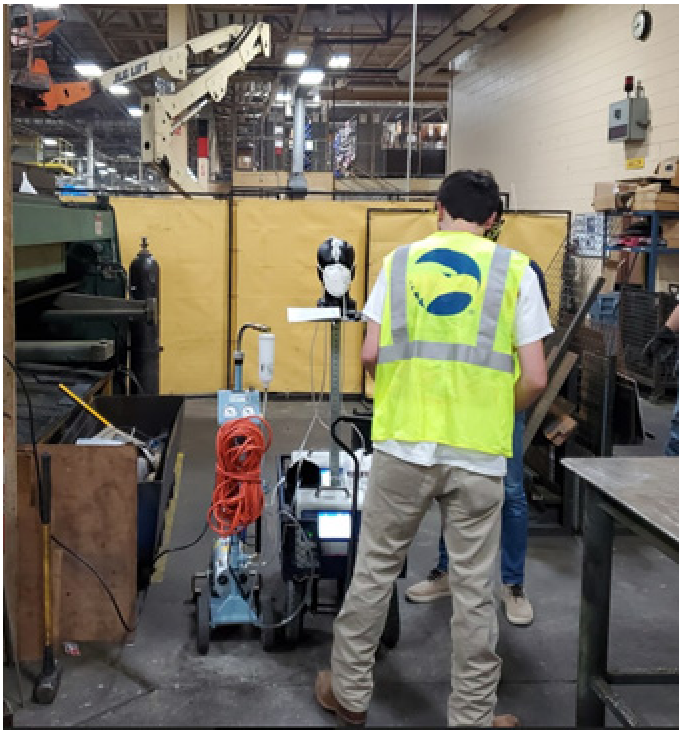

Figure S1. Industrial manufacturing plant with testing setup.

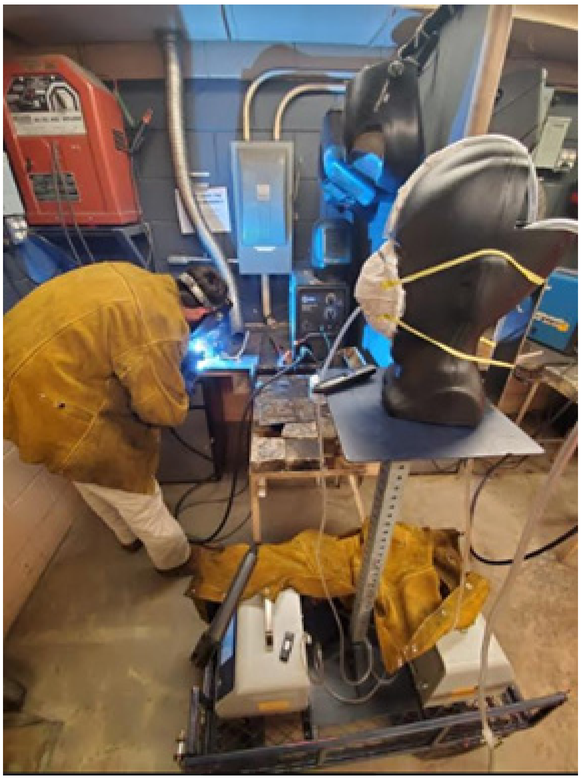

Figure S2. Georgia Southern University Welding Shop with testing setup.

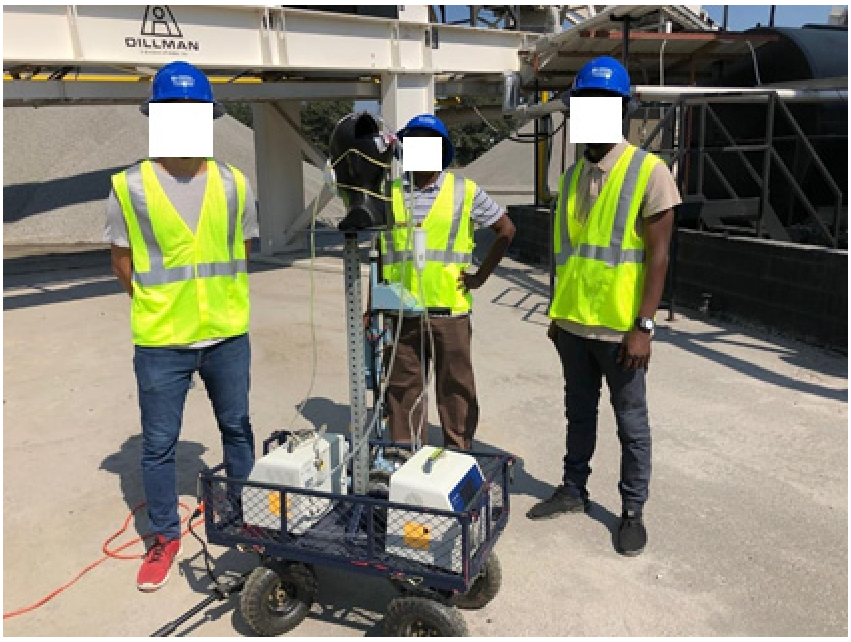

Figure S3. Asphalt Production Plant with testing setup.
